# Co-infection of a hypovirulent isolate of *Sclerotinia sclerotiorum* with a new botybirnavirus and a strain of a mitovirus

**DOI:** 10.1186/s12985-016-0550-2

**Published:** 2016-06-06

**Authors:** Hongchang Ran, Lijiang Liu, Bo Li, Jiasen Cheng, Yanping Fu, Daohong Jiang, Jiatao Xie

**Affiliations:** State Key Laboratory of Agricultural Microbiology, the Provincial Key Lab of Plant Pathology of Hubei Province, College of Plant Science and Technology, Huazhong Agricultural University, Wuhan, 430070 China

**Keywords:** Mycovirus, Botybirnavirus, Mitovirus, Mixed-infection, Hypovirulence, *Sclerotinia sclerotiorum*

## Abstract

**Background:**

*Sclerotinia sclerotiorum*, a notorious plant fungal pathogen, causes yield loss of many crops and vegetables, and is a natural host of a diverse viruses with positive-sense RNA (+ssRNA), negative-sense RNA (−ssRNA), double-stranded RNA (dsRNA), or DNA genomes. Mixed-infection with multiple related or unrelated mycoviruses is a common phenomenon in *S. sclerotiorum*. However, a single strain co-infected with dsRNA and + ssRNA viruses has not been reported in *S. sclerotiorum*.

**Results:**

We report two unrelated viruses, Sclerotinia sclerotiorum botybirnavirus 2 (SsBRV2) with a bipartite dsRNA genome and Sclerotinia sclerotiorum mitovirus 4 (SsMV4/AH16) with a + ssRNA genome, which were originally detected in a single hypovirulent strain AH16 of *S. sclerotiorum*. SsMV4/AH16 has a typical genome of mitovirus and is a strain of mitovirus SsMV4. The genome of SsBRV2 consists of two separated dsRNA segments. The large dsRNA segment is 6159 bp in length and only has a single open reading frame (ORF) encoding a putative 1868-aa polyprotein with a conserved RNA dependent RNA polymerase (RdRp) domain. The small dsRNA segment is 5872 bp in length and encodes a putative 1778-aa protein. Phylogenetic analysis using RdRp conserved domain sequences revealed that SsBRV2 is phylogenetically related to the previously reported three bipartite viruses SsBRV1, Botrytis porri RNA virus 1 (BpRV1), and soybean leaf-associated botybirnavirus 1 (SlaBRV1). Electron microscopy demonstrated that SsBRV2 forms rigid spherical virions with a diameter of approximately 40 nm in infected mycelia. The virion of SsBRV2 was successfully introduced into a virus-free strain, which provides conclusive evidence that SsBRV2 confers hypovirulence on phytopathogenic fungus *S. sclerotiorum*.

**Conclusions:**

A bisegmented dsRNA virus (SsBRV2/AH16) and a nonsegmented + ssRNA virus (SsMV4/AH16) were characterized in a hypovirulent strain AH16 of *S. sclerotiorum*. SsMV4/AH16 is a strain of a reported mitovirus, whereas SsBRV2 is a new botybirnavirus. SsBRV2 is the causal agent of hypovirulence on *S. sclerotiorum*. Our findings supplied a first evidence that a single *S. sclerotiorum* strain is co-infected by dsRNA and + ssRNA mycoviruses.

**Electronic supplementary material:**

The online version of this article (doi:10.1186/s12985-016-0550-2) contains supplementary material, which is available to authorized users.

## Background

Mycovirus, a kind of virus that replicates only inside the cell of fungi or oomycetes [1, 2], was firstly found in a diseased mushroom [3]. Since biological control of chestnut blight with hypovirus (Cryphonectria hypovirus 1) was successfully applied under natural conditions in Europe, an increasing number of mycoviruses have been detected from various phytopathogenic fungi belonging to four phyla of *Chytridiomycota*, *Zygomycota*, *Ascomycota*, and *Basidiomycota* [1, 2]. The phytopathologists are showing more interest in mycoviruses that could reduce the pathogenicity of phytopathogenic fungus and enhance plant resistance to abiotic stress [1, 4–6]. Those debilitation-related mycoviruses have great potential to develop biological agents for virocontrol of plant fungal diseases [1, 2]. So far, more than 300 mycoviruses have been fully or partially sequenced according to the NCBI (National Center for Biotechnology Information) GenBank database, but mycovirus research still significantly lags behind animal or plant virus research.

Mycoviruses usually have double-stranded RNA (dsRNA), single-stranded (ssRNA), and rare ssDNA genomes [4]. Mycoviruses with dsRNA genome are classified into six families, i.e. *Chysoviridae*, *Partitiviridae*, *Reoviridae*, *Totiviridae*, *Megabirnaviridae*, and *Quadriviridae*. Likewise, mycoviruses with ssRNA genome are also classified into six families: *Alphaflexiviridae*, *Barnaviride*, *Endornaviridae*, *Gammaflexiviridae*, *Hypoviridae*, and *Narnaviridae*. With the development of new sequencing methods, more and more mycoviruses have been identified. However, some of the newly identified mycoviruses showed unique molecular and biological properties different from all known mycoviruses, and could not be classified into any of the established virus families. The proposed family Mycomononegaviridae accommodates Sclerotinia sclerotiorum negative-stranded RNA virus 1 (SsNSRV-1) that was a newly reported negative-stranded virus in fungi [4, 7]. A DNA mycovirus, Sclerotinia sclerotiorum hypovirulence-associated DNA virus 1 (SsHADV-1), is the prototype of the proposed family Mycodnaviridae [8]. Two recent reports proposed the establishment of new family Fusariviridae to encompass three previously reported positive-sense RNA mycoviruses: Fusarium graminearum virus 1 (FgV1), Rosellinia necatrix fusarivirus 1 (RnFV1), and Sclerotinia sclerotiorum fusarivirus 1 (SsFV1) [9, 10]. An EC-approved ICTV proposed family Botybirnaviridae (https://talk.ictvonline.org/files/ictv_official_taxonomy_updates_since_the_8th_report/m/fungal-official/5871) comprised three potential members of Botrytis porri botybirnavirus 1 (BpRV1), Sclerotinia sclerotiorum botybirnavirus 1 (SsBRV1), and soybean leaf-associated botybirnavirus 1 (SlaBRV1) [11–13]. These studies have expanded our knowledge of virus genome, taxonomy, evolution and the interaction between viruses and their hosts.

*Sclerotinia sclerotiorum* (Lib.) de Bary is a severe world-wide-spread plant pathogenic fungus and attacks more than 400 plant species [14]. Rapeseed (*Brassica napus*) is a major plant oil crop in China and 85 % of rapeseed is planted in the Yangtze River Basin. However, stem rot disease caused by *S. sclerotiorum*, the most important disease on rapeseed in China, results in a huge loss of production annually. Currently, no resistant cultivar of *B. napus* is available for stem rot disease. While spraying fungicides is an effective method to control stem rot disease at the flowering stage of *B. napus*, the application of fungicides is becoming difficult during flowering time due to the change of the cropping system from transplant to broadcast sowing. Furthermore, chemical fungicide-resistant strains (such as carbendazim resistant strains) were isolated frequently from the field [15]. Screening hypovirulence-associated mycoviruses from *S. sclerotiorum* population and probing their potential as bio-control agents to combat diseases is another potential control strategy for rapeseed stem rot. *S. sclerotiorum* strains are increasingly recognized to harbor great diverse mycoviruses including the newly reported negative-sense RNA mycovirus and DNA mycovirus in fungi [2, 7]. Field experiment supplied multiple lines of evidence that SsHADV-1 as a natural fungicide has a great potential to control rapeseed stem rot [16]. The successful application of SsHADV-1 to the agricultural system has encouraged researchers to screen more strong infective and hypovirulence-associated mycoviruses from *S. sclerotiorum*. Characterization of newly discovered mycoviruses will facilitate our understanding of virus ecology, evolution, and establish mycovirus-*S. sclerotiorum* interaction system at the molecular level.

In the present research, the strain AH16 of *S. sclerotiorum* was identified to have the features of hypovirulence. We isolated and sequenced two unrelated mycoviruses of a mitovirus (Sclerotinia sclerotiorum mitovirus 4, SsMV4) and a botybirnavirus (Sclerotinia sclerotiorum botybirnavirus 2, SsBRV2) in strain AH16. Virion transfection experiment directly indicated that SsBRV2 could be responsible for the hypovirulence of *S. sclerotiorum*.

## Methods

### Fungal strains and culture condition

*S. sclerotiorum* strain AH16 was derived from a sclerotia collected from a diseased rapeseed stem in Anhui province, P.R. China. Strain Ep-1PNA367, a single-ascospore isolate derived from Ep-1PN. Ep-1PNA367R, a strain labeled with a *hygromycin B phosphotransferase* gene by an *Agrobacterium tumefaciens* mediated transformation method, shows no significant difference from its parent strain Ep-1PNA367 in biological properties, and was used as mycovirus transfection recipient strain. All the strains were cultured on potato dextrose agar (PDA) at 20–22 °C and stored at 4 °C.

### RNA isolation and purification

To isolate dsRNA and total RNA from *S. sclerotiorum* strains, mycelia were cultured on PDA plate overlapping cellophane membranes for 3–4 days. The harvested mycelia were ground to fine powder in liquid nitrogen using sterilized mortar and pestle. dsRNA was extracted as previously described [17], and total RNA was extracted with TRIzol Reagent (Invitrogen, CA, USA) according to the manufacturer's instructions. The dsRNA sample was treated with DNase I and S1 nuclease (Takara, Dalian, China), followed by separation by electrophoresis on a 1 % (wt/vol) agarose gel and purification with gel extraction kit (Axygene Biosciences). The gel-purified dsRNA fragments were subjected to sequence analysis. Total RNA was used for cDNA synthesis and PCR analysis.

### Purification of virus particle

Isolation and purification of virus particle were performed as previously described with minor modifications [12, 18]. Briefly, mycelia of SsBRV2-infected strain and SsBRV2-free strain, respectively, were inoculated in conical flasks containing 150 ml PDB and shake-cultured at 200 rpm for 6 days. Next, mycelia were harvested through 4-layer sterilized gauze and then washed several times with sterilized water. After that, the harvested mycelia (30–40 g) were homogenized in the presence of 3 volume phosphate buffer (0.1 M sodium phosphate, pH 7.0, containing 0.2 M KCl and 0.5 % mercaptoethanol) in a Waring blender, followed by centrifugation at 10,000 rpm, 4 °C for 15 min to remove the hyphal cell debris. After two cycles of ultracentrifugation, virus particles were purified with a gradient sucrose concentration of 20–40 % (W/V) and then collected by a new sterile syringe. After ultracentrifugation to remove the sucrose solution, the virus particle pellets were re-suspended in 200 μl sodium phosphate buffer (0.1 M, pH7.0) and the concentration of the final 200 μl of virus particles was determined by spectrometry reading. dsRNA was isolated from the purified viral particles using phenol-chloroform extraction and detected on a 1 % agarose gel. The Structure proteins of the purified viral particles were separated on a 10 % (wt/vol) polyacrylamide gel amended with 1 % (wt/vol) sodium dodecyl sulfate (SDS).

For negative staining, a drop of about 5 μl of the purified virus particle suspension was loaded on the hydrophobic surface of a parafilm square. Several 200-mesh carbon-formvar coated copper grids were floated onto this drop for 5 min and the excess liquid was removed from each of the grids by touching its border with a cut piece of filter paper. Next, the grids loaded with virus particles were immediately re-floated in a drop of 2 % (W/V) phosphotungstic acid solution for 5 min, and the excess virus particle suspension was removed with filter paper and the prepared grids were left to dry for a few minutes. Finally, the prepared samples were visualized under transmission electron microscopy (TEM) as previously described [12].

### Protoplast transfection and transfection assay

The virus-free strain Ep-1PNA367R was used as the recipient strain in the virus transfection experiment. Protoplasts from actively growing mycelia of strain Ep-1PNA367R were prepared as previously described [19]. The purified virus particles were introduced into the protoplasts of Ep-1PNA367R in the presence of polyethylene glycol (PEG) 4000 according to the protocol as previously reported [12]. All five candidate transfectants were further confirmed via dsRNA extraction and RT-PCR amplification with the specific primers for dsRNA1 of SsBRV2 (dsRNA1F: 5'-GCTCTACATCGCTACATTGGTTGG-3', dsRNA1R:5'-GGCTTGTTCATACTCGCACTCTTG-3'), dsRNA2 of SsBRV2 (dsRNA2F: 5'-GGAAAGAGAGCGCAGTGCATACG-3', dsRNA2R: 5'-CCATCCACCCCACCATACTCAG-3'), and mitovirus SsMV4/AH16 (F: 5'-AACAGACCGATTTCCGTTACAA-3' and R: 5'-TTCCACTTACTTCAATACCTCCCT-3').

### cDNA cloning, sequencing and sequence analysis

The partial cDNA sequences of the dsRNA segments isolated from the strain AH16 were obtained as previously described [17, 20]. Terminal sequences were determined as previously described with minor modifications [21]. Briefly, 200–500 ng dsRNA and 30 pmol oligo nucleotide primer PC3-T7 loop (5’-p-GGATCCCGGGAATTCGGTAATACGACTCACTATATTTTTATAGTGAGTCGTATTA–OH-3′) were added into a reaction mixture [50 mM Tris-HCl (pH 7.5), MgCl2 10 mM, DTT 10 mM, 1 mM ATP, 20U RNase inhibitors, 25 % PEG4000 (W/V) and 40 units of T4 RNA ligase] and then ligated at 4–8 °C for 18 h. The ligated product was precipitated and centrifuged for purification. The pellets were re-suspended for cDNA synthesis using cDNA Synthesis Kit according to the manufacturer’s instructions (Fermentas). PCR amplification was performed using the PC2 primer (5’-p-CCGAATTCCCGGGATCC-3’) complementary to the oligonucleotide PC3-T7 loop and the sequence-specific primer corresponding to the 5’-and 3’-terminal sequences of the dsRNA, respectively. The full cDNA sequences of all dsRNA segments were further confirmed by RT-PCR amplification and PCR products were sequenced in two directions. To avoid artificial mistakes, every nucleotide was sequenced at least three times.

The full-length cDNA sequences of dsRNA segments isolated from strain AH16 were initially assembled using DNAMAN software and ORFs were predicted with the ORF finder program (http://www.ncbi.nlm.nih.gov/gorf/orfig.cgi). Domains or motifs were predicted on the motif scan website (http://www.genome.jp/tools/motif/). The protein sequences of selected viruses (Table 1) were then aligned by using multiple sequence alignment software of CLUSTAL-X program and phylogenetic analysis was conducted using MEGA version 6.0 program [22]. The Maximum-Likelihood tree was constructed using the best-fit model (LG + I + G + F) of protein evolution obtained using Akaike’s information criterion (AIC) and searched using the ProtTest server^3^ [23]. Potential RNA secondary structures were predicted and the free energy (ΔG) was estimated using RNA structure software [24].

**Table 1:** The information of all selected viruses used for phylogenetic analysis

Virus Family	Virus name	Abbreviation	Accession Number
*Botybirnaviridae*	Botrytis porri RNA virus 1	BpRV1	YP_006390636.1
	Sclerotinia sclerotiorum botybirnavirus 1	SsBRV1	YP_009141011.1
	soybean leaf-associated botybirnavirus 1	SlaBRV1	ALM62244
*Chrysoviridae*	Agaricus bisporus virus 1	AbV1	CAA64144.1
	Amasya cherry disease associated chrysovirus	ACD-CV	YP_001531163.1
	Helminthosporium victoriae 145S virus	HvV145S	YP_052858.1
	Penicillium chrysogenum virus	PcV	YP_392482.1
*Megabirnaviridae*	Rosellinia necatrix megabirnavirus 1	RnMBV1	YP_003288763.1
	Sclerotinia sclerotiorum megabirnavirus 1	SsMBV1	YP_009143529.1
	Rosellinia necatrix megabirnavirus 2	RnMBV2	YP_009227124
*Quadriviridae*	Rosellinia necatrix quadrivirus 1	RnQV1	BAL46425.1
*Totiviridae*	Coniothyrium minitans RNA virus	CmRV	YP_392467.1
	Gremmeniella abietina RNA virus L1	GaVL1	NP_624332.2
	Helicobasidium mompa No.17 dsRNA virus	Hm17V	NP_898833.1
	Saccharomyces cerevisiae virus L-A	ScV-L-A	AAA50508.1
	Saccharomyces cerevisiae virus L-BC (La)	ScV-L-BC	AAB02146.1
	Trichomonas vaginalis virus 1	TVV1	AED99818.1
	Ustilago maydis virus H1	UmV-H1	NP_620728.1
	Helminthosporium victoriae virus	HvV190S	AAB94791.2
	Leishmania RNA virus 1 - 1	LRV	NP_041191.1
Unclassified	Ustilaginoidea virens nonsegmented virus 1	UvNV1	KJ605397
	Circulifer tenellus virus 1	CiTV1	YP_003800003.1
	Cucurbit yellows-associated virus	CYAV	CAA63099.2
	Phytophthora infestans RNA virus 3	PiRV3	AEX87902.1
	Diplodia scrobiculata RNA virus 1	DsRV1	YP_003359178.1
	Fusarium graminearum dsRNA mycovirus-3	FgV3	YP_003288789.1
	Lentinula edodes mycovirus HKB	LeV-HKB	BAG71788.2
	Persimmon latent virus	PLV	YP_009025166.1
	Phlebiopsis gigantea mycovirus dsRNA 1	PgRV1	CAJ34333.2
	Spissistilus festinus virus 1	SpFV1	YP_003800001.1
	Phlebiopsis gigantea mycovirus	PgRV2	CAJ34335.2

### Assay of biological properties of mycovirus-infected and mycovirus-free strains

To define whether mycoviruses were responsible for phenotypic change between mycovirus-infected and mycovirus-free strains of *S. sclerotiorum*, the biological features including colony morphology, growth rate and virulence were evaluated. Colony development of individual strains was observed on PDA plates in succession and recorded separately with a camera at 3 days-post-inoculation (dpi), 7 dpi, and 15 dpi. To obtain the growth rate, all actively growing strains were cultured on fresh PDA plate and the diameter of colonies was measured separately at 24 and 48 h post-inoculation (hpi). The growth rate was then calculated using the method previously described [19]. The pathogenicity assay of *S. sclerotiorum* strains was performed on the detached soybean leaves as previously described [25]. All assays were repeated three times. Experimental data were analyzed by the SAS 8.0 program. Treatment means were compared using the least significant difference test at *P* = 0.05 level.

## Results and Discussion

### Visualization of three dsRNA segments in the strain AH16 of *S. sclerotiorum*

**Fig. 1:**
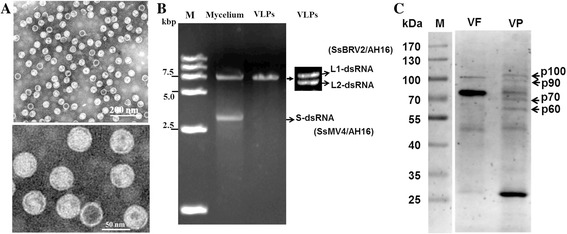
Virus particles and dsRNA components in strain AH16. **a** Electron micrograph of SsBRV2 virus particles purified from the mycelia of strain AH16. **b** Electrophoresis on 1 % agarose gel of dsRNA extracted from mycelia and purified VLPs (left figure), and dsRNA segments isolated from the purified particles were further separated on 5 % PAGE gel (right figure). All of the dsRNA samples were treated with DNase I and S1 nuclease prior to electrophoresis. M means DNA molecular weight; kbp means kilo base pair. **c** 10 % SDS-PAGE gel-electrophoresed analysis of the protein components of viral particles purified from SsBRV2-infected strain (lane VP) and SsBRV2-free strain (lane VF). Four black arrows representative protein bands that were presence in SsBRV2-infected strain, but lack in SsBRV2-free strain. The molecular weight of the protein bands was estimated by the protein markers. kDa means kilo Dalton

*S. sclerotiorum* strain AH16 shows abnormal phenotypes with less sclerotia and lower virulence on its host, which was similar to the phenotypes of other previously reported mycovirus-infected strains of *S. sclerotiorum* [26]. Thus we attempted to purify the virus-like particles (VLPs) with sucrose density gradient (20–40 %) centrifugation. The purified VLPs were further observed by TEM. Consistent with previous reports [11, 12, 27], VLPs were successfully extracted from the mycelia of the strain AH16. TEM observation suggested that these VLPs have morphological features of rigid spherical particles with a diameter of approximately 40 nm (Fig. 1a). Nucleic acids were extracted from these purified particles, showing that the purified VLPs accommodate a dsRNA segment (L-dsRNA) resistant to S1 nuclease and DNase I. When L-dsRNA elements were resolved on 5 % PAGE gel for 48 h, two similar sized L-dsRNA segments (L1-dsRNA and L2-dsRNA) were obviously separated (Fig. 1b, right figure), revealing that purified VLPs contain at least 2 dsRNA species, and strain AH16 is infected by one or more mycoviruses.

Previous studies reported that some mycoviruses (such as mitovirus and hypovirus) lack the true virion in their life cycles [28, 29]. To clarify whether strain AH16 harbors mycoviruses lacking the true virion, dsRNA and extra-chromosome DNA elements were directly extracted from the mycelia of strain AH16 using CF-11 cellulose chromatography and the CTAB method, respectively. Agarose electrophoresis and PAGE detection results indicated that three dsRNA segments (two L-dsRNA and one S-dsRNA) co-infected the strain AH16, but extra-chromosome DNA elements failed to be obtained. Two L-dsRNA segments from the mycelia had the same migration rate with that released from VLPs on agarose gel (Fig. 1b). Then, we subsequently demonstrated that the two L-dsRNA segments from the mycelia were associated with the purified VLPs, and further confirmed that they represented the genome of a new botybirnavirus (temporarily named Sclerotinia sclerotiorum botybirnavirus 2, SsBRV2/AH16), whereas S-dsRNA was the genome of a mitovirus (temporarily named Sclerotinia sclerotiorum mitovirus 4, SsMV4/AH16). Therefore, strain AH16 carries at least two mycoviruses, one with two dsRNA segments, the other with a single ssRNA segment.

The purified virus particles from SsBRV2-infected strain and SsBRV2-free strain, respectively, were further separated on SDS-PAGE gel-electrophoresed analysis (Fig. 1c). The results showed that four protein components with protein size of p100, p90, p70 and p60 were presence in SsBRV2-infected strain (lane VP), but lack in SsBRV2-free strain (lane VF). Therefore, SsBRV2 is comprised of four structure proteins.

### SsMV4/AH16 is a new strain of Sclerotinia sclerotiorum mitovirus 4

**Fig. 2:**
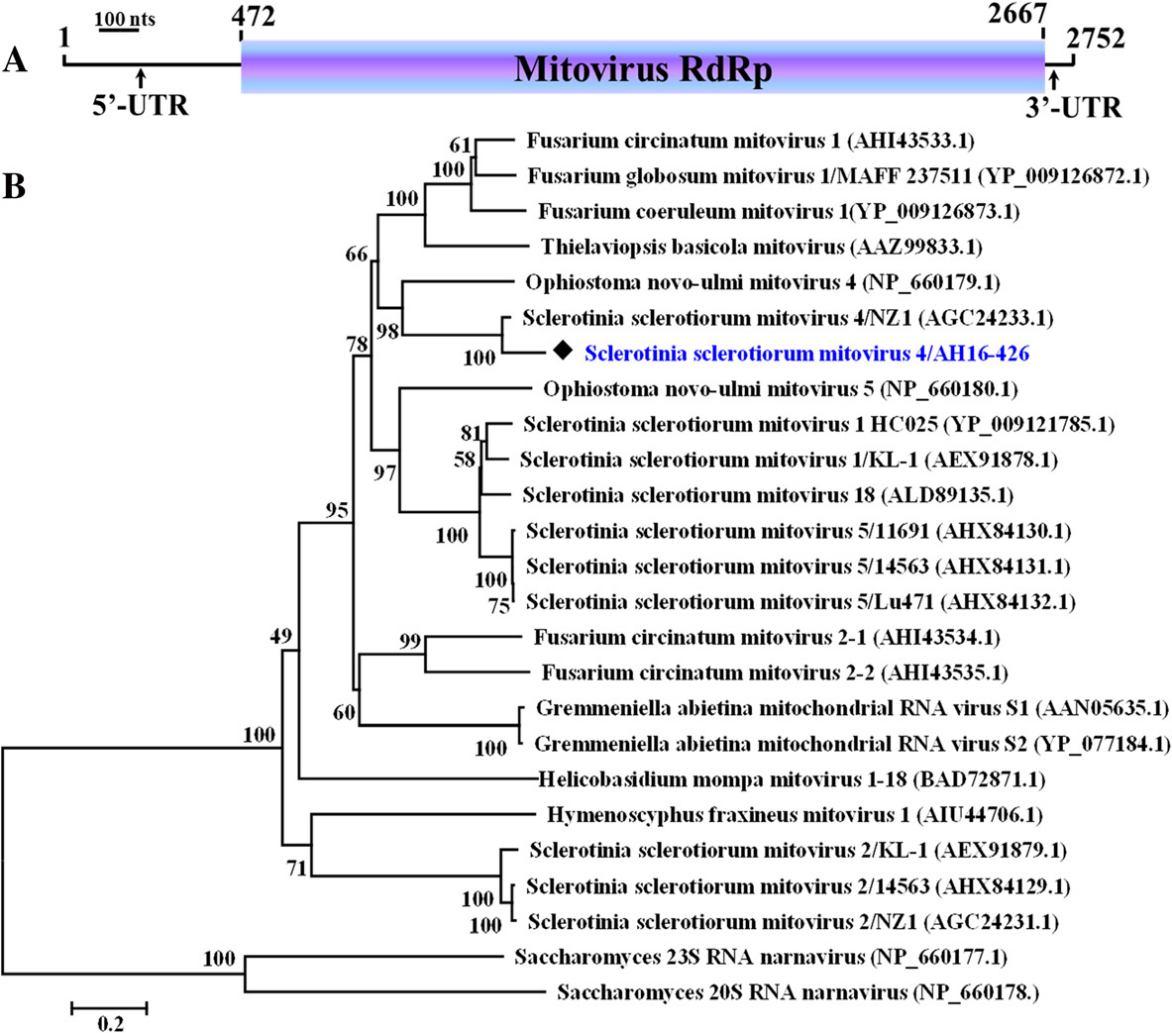
A schematic representation and phylogenetic analysis of SsMV4/AH16 from strain AH16. **a** Diagrammatic representation of the genomic organization of SsMV4/AH16 indicating the presence of a single large ORF. **b** Maximum likelihood (ML) phylogenetic tree based on multiple alignments of RdRp regions of SsMV4/AH16 and other mitoviruses. The bootstrap values (%) obtained with 1000 replicates is indicated on the branches. Scale bar at lower left corresponds to a genetic distance of 0.2. The accession number in parentheses is followed by the virus name

The genetic organization of SsMV4/AH16 is shown in Fig. 2 and the complete nucleotide sequence of SsMV4/AH16 was deposited in the GenBank database under the accession number of KT962974. Similar to the size (~2.7 kb) estimated by agarose gel electrophoresis, the genome of SsMV4 comprises 2752 nucleotides with a low GC content of 31 %. The 5’ and 3’- UTRs are 471 and 85 nts long, respectively. SsMV4/AH16 contains a single ORF, and encodes a protein of RNA-dependent RNA polymerase (RdRp) when fungal mitochondrial code was applied (Fig. 2a). Multiple alignment and BLAST search revealed that SsMV4/AH16 RdRp shares high sequence identity (93 %) with a strain (SsMV4/NZ1) of Sclerotinia sclerotiorum mitovirus 4. Phylogenic analysis further supported that SsMV4/AH16 is closely related phylogenetically to members of the families *Narnaviridae* (Fig. 2b). Based on the ICTV rules of species demarcation criteria about mitovirus, strains of the same mitovirus species should share greater than 90 % identity with each other [28, 30]. Thus, SsMV4/AH16 and SsMV4/NZ1 belong to the same species (Sclerotinia sclerotiorum mitovirus 4) in genus *Mitovirus*. Previous reports proved that mitovirus is rich in diversity of strains in the population of *S. sclerotiorum*. Thus far, thirteen mitovirus strains belonging to seven mitovirus species (SsMV1 to SsMV7) have been characterized in *S. sclerotiorum*. SsMV1/KL-1 and SsMV2/KL-1 were isolated from a USA strain [17]. SsMV1/HC025 infected a Chinese strain [25]. Strains of SsMV2 to SsMV7 were discovered in New Zealand [31, 32]. Here, a new mitovirus strain SsMV4/AH16 was reported in China. These reports suggested that *S. sclerotiorum* isolates are commonly infected by mitoviruses regardless of their geographical origin.

### SsBRV2, a bipartite dsRNA virus, is related phylogenetically to botybirnavirus

**Fig. 3:**
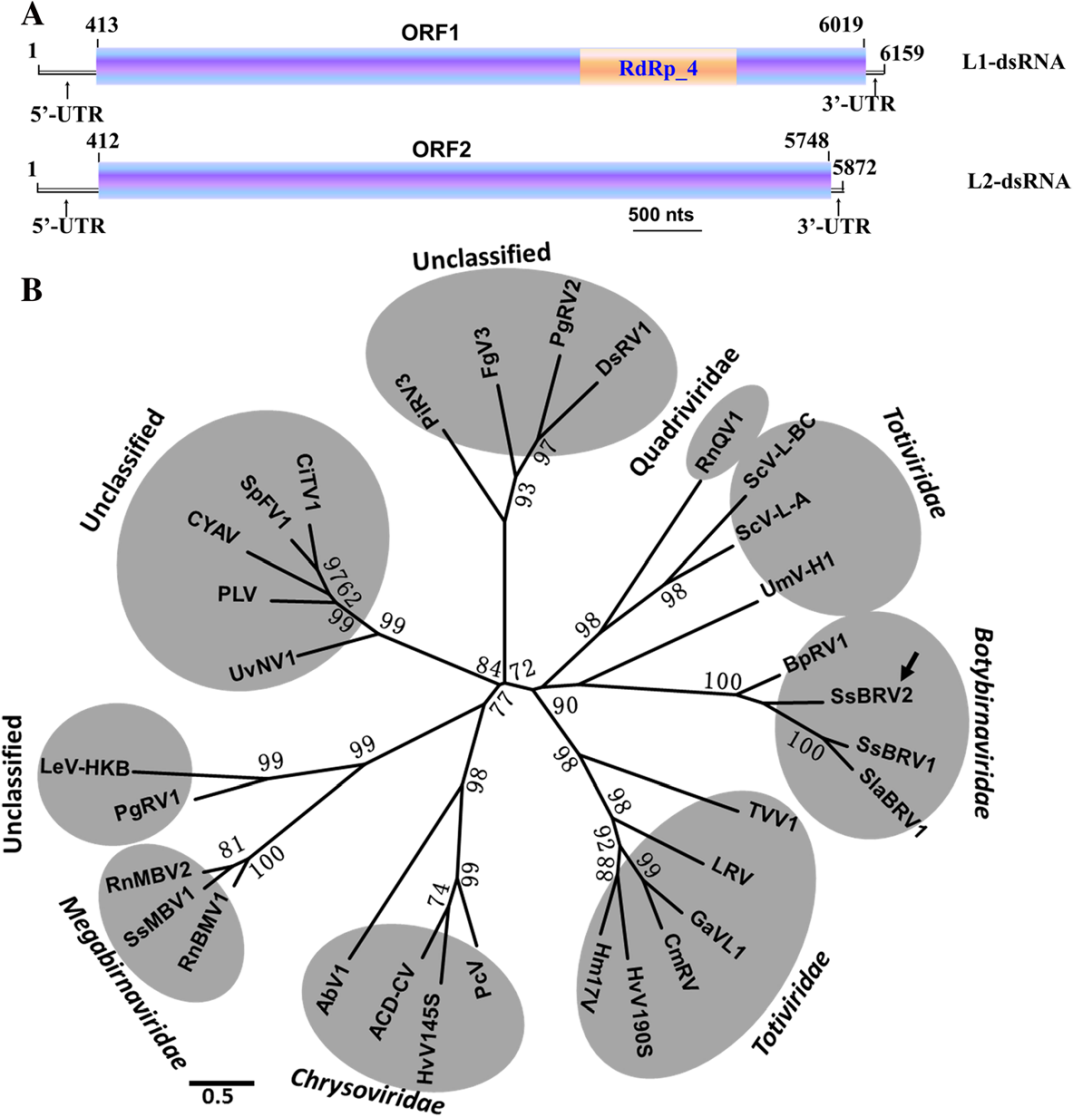
Genetic organization and phylogenetic analysis of the virus SsBRV2. **a** Schematic representation of the genetic organization of SsBRV2. Two ORFs are shown as an open box and locate on the positive-strand of L1 and L2-dsRNA, respectively. A conserved domain of RNA-dependent RNA polymerase (RdRp) of ORF1-encoded putative protein is highlighted by yellow shading. **b** An phylogenetic tree constructed based on an alignment of respective RdRp conserved domain amino acid sequences of selected viruses. SsBRV2 was marked with a black arrow. Bootstrap values (%) obtained with 1000 replicates are indicated on branches and branch lengths correspond to genetic distance; scale bar at lower left corresponds to a genetic distance of 0.5. All information of the selected viruses in phylogenetic tee was shown in Table 1

SsBRV2 has a bipartite genome consisting of L1-dsRNA and L2-dsRNA (Fig. 3). The complete genome of SsBRV2 was first determined using the method of tagged-random PCR. Fifty-eight random cDNA clones were obtained and randomly matched in different positions of two dsRNA segments. The gaps between different clones were generated by specific PCR, and terminal sequences of two dsRNA segments were determined from RACE clones. The full-length cDNA sequences of L1-dsRNA and L2-dsRNA were found to be 6159 and 5872 bp, respectively (Fig. 3), which were deposited in NCBI database under the accession numbers of KT962972 (for L1-dsRNA) and KT962973 (for L2-dsRNA).

ORFs were analyzed from the sequences of two segments, revealing that they were homologous to the corresponding segments of three other botybirnaviruses. ORF1, starting at nt position 413 and terminating at nt 6019, was identified on the positive strand of L1-dsRNA, and was deduced to encode a 1868-aa protein (209 kDa) (Fig. 3a). This deduced ORF1-encoded protein contains an RdRp (RdRp_4 superfamily, E-value = 1.9e-32) domain with eight conserved motifs (I to VIII) (Additional file 1: Figure. S1), which was similar to the RdRp sequences of the previous reports (11, 12). Multiple alignment based on the RdRp conserved domain revealed that SsBRV2 was phylogenetically related to previously reported SsBRV1 (46 % identities), SlaBRV1 (38 % identities), BpRV1 (55 % identities). The phylogenetic tree also indicated that SsBRV2 formed a well-supported clade with SsBRV1, SlaBRV1 and BpRV1, which was distant from other known dsRNA mycoviruses (Fig. 3b). ORF2 detected on the positive strand of L2-dsRNA codes for a putative 1778-aa protein with unknown functions (145 kDa) (Fig. 3a). Although no putative conserved domains were predicted in ORF2-encoded protein, the region (position from Gly184 to Arg1146) of the ORF2-encoded protein shares low sequence identity to the corresponding regions of SsBRV1 (27 %) and BpRV1 (25 %). Similar to other botybirnaviruses [11, 12], SsBRV2 has a long 5’-UTR but a relatively short 3’- UTR, and the terminal sequences of the two dsRNA segments of SsBRV2 are conserved with a sequence identity of 82.3 % (5’ terminal region) and 98.7 % (3’ terminal region) (Additional file 2: Figure. S2). The 5’-UTRs and 3’-UTRs of SsBRV2 were detected to form stable secondary structures with a ΔG value of −13.3 kcal/mol and −23.0 kcal/mol, respectively (Additional file 3: Figure S3).

The aforementioned results indicated that SsBRV2, the second botybirnavirus in *S. sclerotiorum* following the first reported botybirnavirus SsBRV1 in the strain SCH941 [12], is a novel bipartite dsRNA mycovirus belonging to the newly proposed family Botybirnaviridae. Although SsBRV1, SsBRV2, SlaBRV1, and BpRV1 are phylogenetically related with each other, there are five obvious differences among the four botybirnaviruses. First, three botybirnaviruses of SsBRV1, SsBRV2, and BpRV1 infect filamentous fungi [11, 12], whereas SlaBRV1 was detected from soybean phyllosphere via a metatranscriptomics technique and its complete genome was partially obtained [13]. Second, a satellite-RNA associated SsBRV1 was discovered in its fungal host [12], whereas no similar satellite-RNA was detected in SsBRV2-infected strains. Third, a GHBP domain (animal growth hormone receptor binding domain) was identified in SsBRV1 ORF2-encoded protein [12], which was not detected in that of SsBRV2 and BpRV1. Fourth, the first start codons (ATG) of ORF1 and ORF2 are located in the strictly conserved region of 5’ terminal of SsBRV2 and BpRV1 [11], whereas the strictly conserved region of SsBRV1 is located inside 5’-UTR [12]. Finally, SsBRV1 co-infected *S. sclerotiorum* strain SCH941 with an unpublished dsRNA mycovirus (a reovirus) [12], whereas SsBRV2 co-infected strain AH16 with a + ssRNA mycovirus (mitovirus). The phenomenon that a single strain was naturally co-infected by dsRNA and + ssRNA have not been reported in *S. sclerotiorum*.

### SsBRV2 is associated with hypovirulence on *S. sclerotiorum*

To confirm whether SsBRV2 or/and SsMV4 is responsible for the hypovirulence of strain AH16, three approaches (virus horizontal transmission, protoplast regeneration isolation, and VLP transfection) were attempted as previously reported [12, 18]. Both mycoviruses (SsBRV2 or/and SsMV4) failed to transmit horizontally from strain AH16 to Ep-1PNA367R via hyphal contact, since strain AH16 is vegetatively incompatible with Ep-1PNA367R. To eliminate two mycoviruses from strain AH16, protoplasts were prepared and 38 protoplast regenerants were isolated. However, RT-PCR and dsRNA extraction confirmed that all the obtained protoplast regenerants still harbored two mycoviruses of SsBRV2 and SsMV4.

**Fig. 4:**
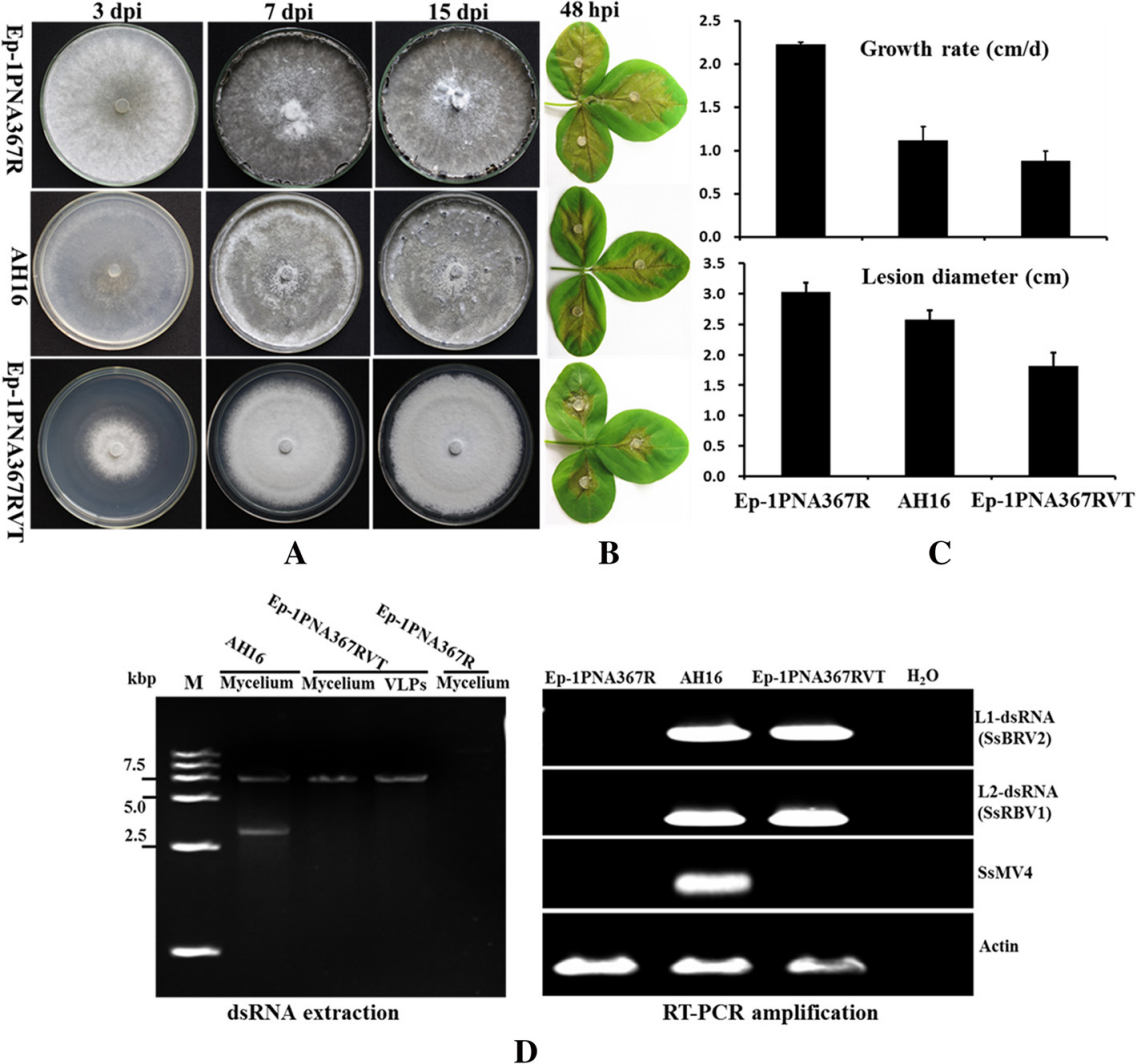
SsBRV2 confers hypovirulence to *S. sclerotiorum*. Colony morphology (**a**) and growth rate (**c**) of the virus-free strain Ep-1PNA367R, virus (SsBRV2 and SsMV4)-infected strain AH16, and SsBRV2 transfectant Ep-1PNA367RVT were recorded at 3 dpi (days post-inoculation), 7 dpi and 15 dpi at 20 °C. **b** Virulence assay of the three strains on the detached soybean leaves, and **c** Lesion diameter calculated at 24 hpi (hours post-inoculation) at 20 °C, 100 % humidity. **d** dsRNA contents and RT-PCR detection of SsBRV2 and SsMV4 in strains Ep-1PNA367R, AH16 and Ep-1PNA367RVT. The actin gene of *S. sclerotiorum* was used as an internal control

We successfully introduced the purified SsBRV2 VLPs into the virus-free strain Ep-1PNA367R of S*. sclerotiorum* and the transfectants of SsBRV2 were confirmed by repeated sub-culturing to be stable in phenotype and virus composition. One (Ep-1PNA367RVT) of the five transfectants were used for biological feature comparison. The results based on dsRNA extraction, or RT-PCR with the SsBRV2 and SsMV4-specific primers revealed that Ep-1PNA367RVT carries SsBRV2 but lacks SsMV4/AH16 (Fig. 4d). Compared to virus-free strain Ep-1PNA367R, strain AH16 has a slower growth rate (1.06 cm/d vs 2.24 cm/d) (Fig. 4c), and caused smaller lesions on detached soybean leaves (Fig. 4b). Strain Ep-1PNA367R formed sclerotia at 7dpi, whereas strain AH16 did not produce sclerotia at this time, and formed fewer, smaller sclerotia at 15 dpi. Interestingly, compared with strain AH16, Ep-1PNA367RVT showed more obvious hypovirulence phenotypic traits including lower growth rate, less virulence and no sclerotial production (Fig. 4a). Moreover, SsBRV2 could transmit from Ep-1PNA367RVT to virus-free strain Ep-1PNA367 and the newly SsBRV2-infected strain exhibited a subset of phenotypic traits similar to those of Ep-1PNA367RVT. Therefore, by transfecting virus-free *S. sclerotiorum* protoplasts with purified virus particles, we obtained unequivocal evidence that SsBRV2 confers hypovirulence to *S. sclerotiorum*.

VLPs transfection experiments suggested that BpRV1 was the causal agent of hypovirulence in *Botrytis porri*, whereas SsBRV1 without satellite-like RNA had latent infection in *S. sclerotiorum* [12]. Our present results indicated clearly that SsBRV2 is related to hypovirulence on *S. sclerotiorum* and they also revealed the diversity of the interactions between botybirnavirus and its host fungi. As for the question why Ep-1PNA367RVT infected with SsBRV2 alone exhibits more seriously debilitating symptoms than strain AH16 co-infected SsBRV2 and SsMV4, it could be answered from the following two aspects. First, the genetic background is different between strain Ep-1PNA367 and AH16, and the unknown interaction mechanism could be involved between SsBRV2 and the two different *S. sclerotiorum* strains. Secondly, similar to interference among unrelated RNA viruses in filamentous fungus *Cryphonectria parasitica* [33], the two unrelated mycoviruses of SsBRV2 and SsMV4 may undergo antagonistic interactions in a single isolate of *S. sclerotiorum*. A previous report suggested that SsMV4/NZ1 contributes a limited effect on the hypovirulence of *S. sclerotiorum* [31]. Whether SsMV4/AH16 is a contributor to phenotypic change of *S. sclerotiorum* needs to be further elucidated.

## Conclusions

In the current study, a bisegmented dsRNA virus (SsBRV2/AH16) and a nonsegmented +ssRNA virus (SsMV4/AH16), which co-infects a single strain AH16 of *S. sclerotiorum,* were characterized. Based on biological characteristics (genome size and organization, etc.) and phylogenetic analysis, SsMV4/AH16 is a new strain of the proposed genus Sclerotinia sclerotiorum mitovirus 4, whereas SsBRV2 is closely related to two previously reported botybirnaviruses, SsBRV1 and BpRV1, which belong to the recently proposed family Botybirnaviridae. The successful introduction of purified VLPs of SsBRV2 into a virus-free isolate of *S. sclerotiorum* directly confirmed that SsBRV2 is the causal agent of hypovirulence. Our findings also supplied a first evidence that a single S. sclerotiorum strain is co-infected by dsRNA and +ssRNA mycoviruses.

## Additional files

Additional file 1: Figure S1. Multiple alignments of conserved RdRp amino acid motifs of SsBRV2 and its relation viruses. Eight motifs (I–VIII) were shown by black lines above RdRp conserved motif sequences. Asterisks, colons and dots represent identical residues, conserved and semi-conserved amino acid residues, respectively. The number of amino acid residues of two separating the motifs is shown in square brackets. (PDF 121 kb) Additional file 2: Figure S2. The 5’- (A) and 3’ - (B) terminus sequence alignment of the two L-dsRNA segments. (PDF 384 kb) Additional file 3: Figure S3. A potential stable stem-loop structure in the 5’-terminal sequence (left) and a triple stem-loop structure in 3’-terminal sequences (right) were predicted with a RNA structure software. (PDF 60 kb)
