# Supplementary material for: Co-infection of a hypovirulent isolate of Sclerotinia sclerotiorum with a new botybirnavirus and a strain of a mitovirus
Source: Virol J. 2016 Jun 6;13:92. doi: 10.1186/s12985-016-0550-2 (PMC4895950; doi:10.1186/s12985-016-0550-2)
Supplement: Additional file 1: Figure S1. — Multiple alignments of conserved RdRp amino acid motifs of SsBRV2 and its relation viruses. Eight motifs (I–VIII) were shown by black lines above RdRp conserved motif sequences. Asterisks, colons and dots represent identical residues, conserved and semi-conserved amino acid residues, respectively. The number of amino acid residues of two separating the motifs is shown in square brackets. (PDF 121 kb) [file 12985_2016_550_MOESM1_ESM.pdf]

```
            Motif I  Motif II           Motif III               Motif IV          Motif V
SsBRV2    LVGRP(67)WM-IGGS(59)KGNE-NG-KL—RAIYGSLFSQY(47)LDYTDFNASHS(55)KGRGEYLRL(09)
SsBRV1    FVGRP(67)WM-IKGS(59)KGQE-NG-KI—RSIQGSCYSHY(48)ADYPDFGATHS(55)SISSEYLRE(08)
SlaBRV1   LVGRP(67)WM-IKGS(59)KGQE-NA-KI—RSIQASLYSHY(48)LDYPDFGATHS(55)RMGSEYLRE(09)
BpRV1     MVGRP(67)WM-IKGS(59)KGNE-HG-KV—RAIYGSLYAHY(47)LDYADFNAQHS(55)QGKGEYLRL(09)
ScV-L-A   LMNRG(60)WV-PGGS(49)KYEW--G-KQ—RAIYGTDLRST(44)FDYDDFNSQHS(52)-SISEFLRV(12)
UmV-H1    LYGRG(66)WL-VSGS(58)KLNETGG-KA—RAIYGVTLWHY(47)YDYPDFNSMHT(64)HKHHEYLRI(09)
HvV145S   LLGRR(73)WM-TKGS(61)KLNENGH-KD—RVLLPGGLLHY(44)YDWANFNVQHS(49)-RNSEFFRV(08)
RnMBV1    ALGRF(72)FG-SSGS(46)KREA--G-KL—RQLLPGRIPHW(48)VDWADFNITHT(69)GEMGEFLRH(08)
SpFV1     LTGRS(91)WI-ASGS(58)KYEN--G-KA—RAIYGVEPMHY(49)LDYADFNRHHT(53)PGRGEYLRV(08)
            .*     :    **    *       *  * :             *: :*.  *.        *.**

               Motif VI               Motif VII              Motif VIII
SsBRV2    GLFSGLPQTTFDNTVANLTYD(19)YVLGDDGWVSF(18)ETNEIKQ-----LIS GSPLRALANIC
SsBRV1    GMFSGVVQTTLINTVMNGALR(19)FELGDDGWAEF(18)ELNSLKQ-----LIS GCASRALAMLV
SlaBRV1   GMFSGVVQTTLFNTVLNGALR(19)YELGDDGWALF(18)QLNPLKQ-----LVS GCPLRALAMLV
BpRV1     GMFSGVRQTTLINTILNLTYH(19)YVLGDDGWVEF(18)EINAIKQ-----LIG GCPVRSLASFV
ScV-L-A   TLLSGWRLTTFMNTVLNWAYM(14)VHNGDDVMISL(18)RAQPAKC-----NLF QYLSRSCATLV
UmV-H1    GLYSGDRDTTLINTLLNIAYA(19)LCHGDDIITVH(18)KGQESKL-----MID)GCLARCVATYV
HvV145S   GLYSGWRGTTWDNTVLNGCYM(19)DQGGDDVDQEF(18)EATKSKQ-----MIG ASPVRGLATFV
RnMBV1    GLWSGWRTTSFINCSFNVAYC(19)VHAGDDFFGTY(18)EVNAQKQ-----LVG GSVMRSIGSFV
SpFV1     GMFSGTRSTDLINTLLNLAYF(20)VHQGDDVWVSN(18)IFQGSKQ-----MFG GYFARSLANYL
           : **   *   *   *           ***              *              *
```
