# Supplementary material for: Co-infection of a hypovirulent isolate of Sclerotinia sclerotiorum with a new botybirnavirus and a strain of a mitovirus
Source: Virol J. 2016 Jun 6;13:92. doi: 10.1186/s12985-016-0550-2 (PMC4895950; doi:10.1186/s12985-016-0550-2)
Supplement: Additional file 2: Figure S2. — The 5’- (A) and 3’ - (B) terminus sequence alignment of the two L-dsRNA segments. (PDF 384 kb) [file 12985_2016_550_MOESM2_ESM.pdf]

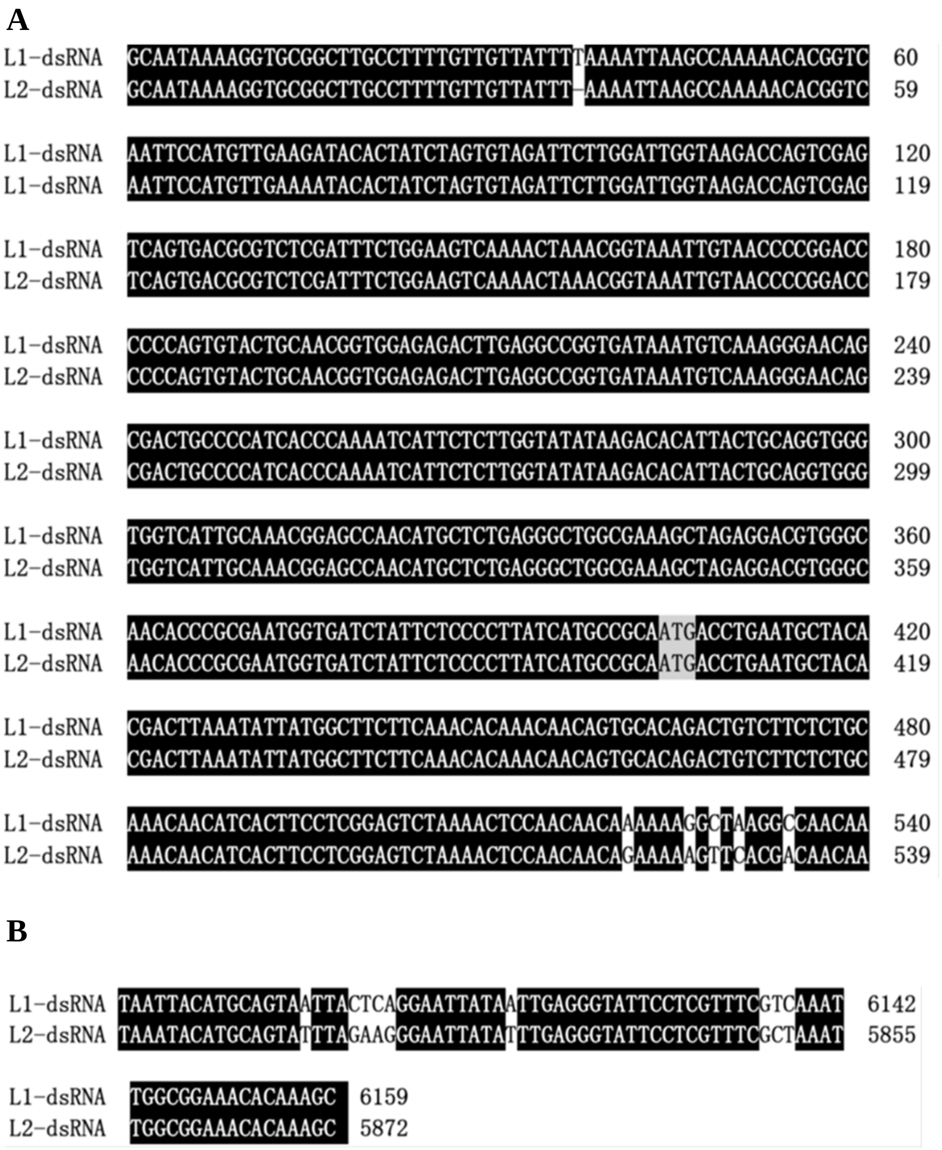
A
L1-dsRNA GCAATAAAAGGTGCGGCTTGCCTTTTGTTGTTATTTTAAAATTAAGCCAAAAACACGGTC 60
L2-dsRNA GCAATAAAAGGTGCGGCTTGCCTTTTGTTGTTATTT-AAAATTAAGCCAAAAACACGGTC 59
L1-dsRNA AATTCCATGTTGAAGATACACTATCTAGTGTAGATTCTTGGATTGGTAAGACCAGTCGAG 120
L1-dsRNA AATTCCATGTTGAAAATACACTATCTAGTGTAGATTCTTGGATTGGTAAGACCAGTCGAG 119
L1-dsRNA TCAGTGACGCGTCTCGATTTCTGGAAGTCAAAACTAAACGGTAAATTGTAACCCCGGACC 180
L2-dsRNA TCAGTGACGCGTCTCGATTTCTGGAAGTCAAAACTAAACGGTAAATTGTAACCCCGGACC 179
L1-dsRNA CCCCAGTGTACTGCAACGGTGGAGAGACTTGAGGCCGGTGATAAATGTCAAAGGGAACAG 240
L2-dsRNA CCCCAGTGTACTGCAACGGTGGAGAGACTTGAGGCCGGTGATAAATGTCAAAGGGAACAG 239
L1-dsRNA CGACTGCCCCATCACCCAAAATCATTCTCTTGGTATATAAGACACATTACTGCAGGTGGG 300
L2-dsRNA CGACTGCCCCATCACCCAAAATCATTCTCTTGGTATATAAGACACATTACTGCAGGTGGG 299
L1-dsRNA TGGTCATTGCAAACGGAGCCAACATGCTCTGAGGGCTGGCGAAAGCTAGAGGACGTGGGC 360
L2-dsRNA TGGTCATTGCAAACGGAGCCAACATGCTCTGAGGGCTGGCGAAAGCTAGAGGACGTGGGC 359
L1-dsRNA AACACCCGCGAATGGTGATCTATTCTCCCCTTATCATGCCGCAATGACCTGAATGCTACA 420
L2-dsRNA AACACCCGCGAATGGTGATCTATTCTCCCCTTATCATGCCGCAATGACCTGAATGCTACA 419
L1-dsRNA CGACTTAAATATTATGGCTTCTTCAAACACAAACAACAGTGCACAGACTGTCTTCTCTGC 480
L2-dsRNA CGACTTAAATATTATGGCTTCTTCAAACACAAACAACAGTGCACAGACTGTCTTCTCTGC 479
L1-dsRNA AAACAACATCACTTCCTCGGAGTCTAAAACTCCAACAACAAAAAAGGCTAAGGCCAACAA 540
L2-dsRNA AAACAACATCACTTCCTCGGAGTCTAAAACTCCAACAACAGAAAAAGTTCACGACAACAA 539
B
L1-dsRNA TAATTACATGCAGTAATTACTCAGGAATTATAATTGAGGGTATTCCTCGTTTCGTCAAAT 6142
L2-dsRNA TAAATACATGCAGTATTTAGAAGGGAATTATATTTGAGGGTATTCCTCGTTTCGCTAAAT 5855
L1-dsRNA TGGCGGAAACACAAAGC 6159
L2-dsRNA TGGCGGAAACACAAAGC 5872
