# Supplementary figures and images for: Co-infection of a hypovirulent isolate of Sclerotinia sclerotiorum with a new botybirnavirus and a strain of a mitovirus
Source: Virol J. 2016 Jun 6;13:92. doi: 10.1186/s12985-016-0550-2 (PMC4895950; doi:10.1186/s12985-016-0550-2)

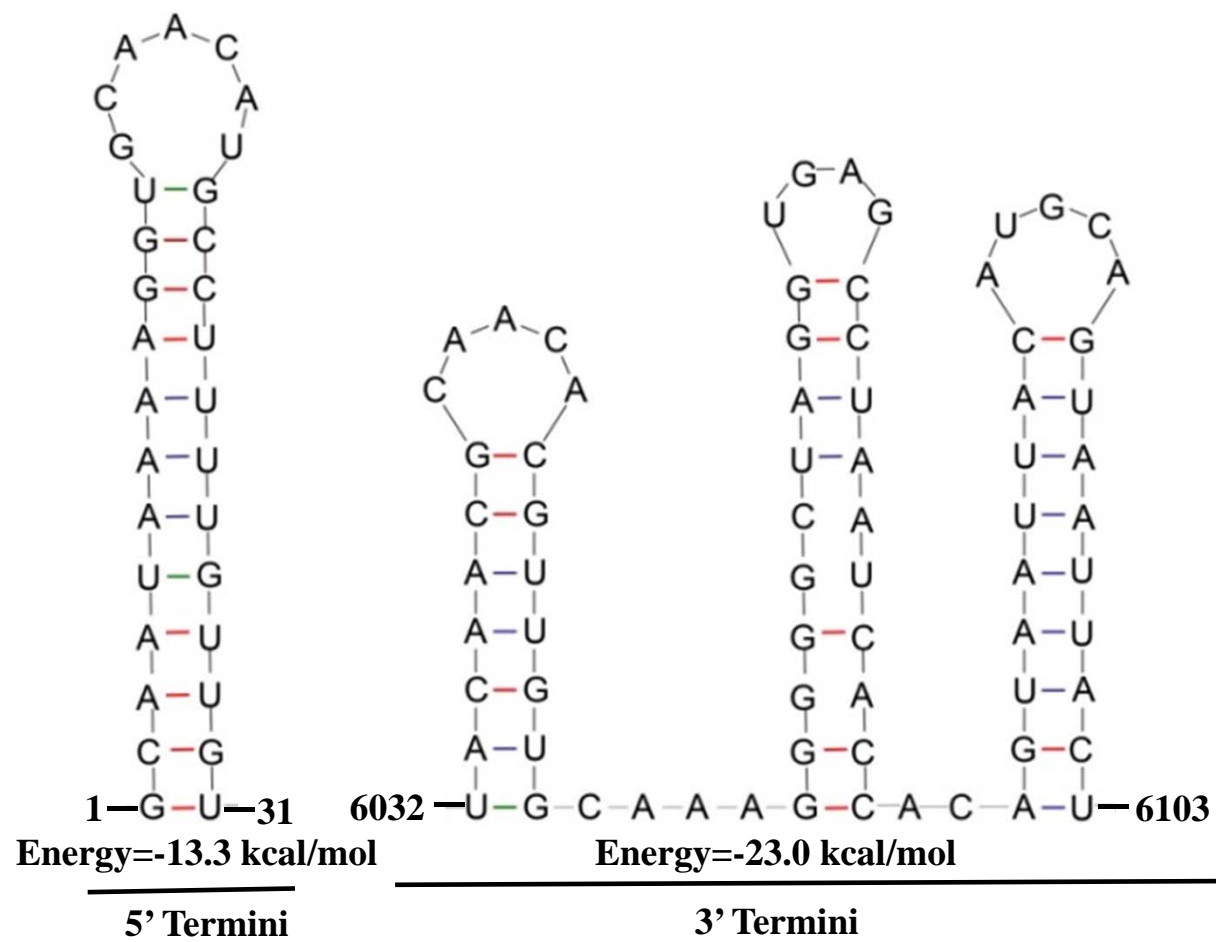

Supplement: Additional file 3: Figure S3. — A potential stable stem-loop structure in the 5’-terminal sequence (left) and a triple stem-loop structure in 3’-terminal sequences (right) were predicted with a RNA structure software. (PDF 60 kb) [file 12985_2016_550_MOESM3_ESM.pdf]
